# Cutaneous Tuberculosis Presenting As Chronic Non-healing Foot Ulcer for 15 Years: A Case Report

**DOI:** 10.7759/cureus.104985

**Published:** 2026-03-10

**Authors:** Ali M Al-Jaafari, Nasser S Al-Farsi, Fatema H Al-Sinaidi

**Affiliations:** 1 Dermatology, Jalan Polyclinic, Jalan Bani Bu Ali, OMN

**Keywords:** cutaneous tuberculosis, misdiagnosis, non-healing ulcer, skin infection, tuberculosis

## Abstract

Cutaneous Tuberculosis is a rare form of extra-pulmonary tuberculosis. It is a skin infection typically caused by *Mycobacterium tuberculosis*. It is more prevalent in TB-endemic areas and immunocompromised individuals. Cutaneous TB has various manifestations and can resemble a multitude of other skin conditions. The disease can be further classified by the route of infection and the bacillary load, which adds another layer of complexity to the diagnostic process. Awareness of cutaneous TB can prompt earlier initiation of microbiological and histopathological workup for the disease, thereby preventing complications and facilitating more effective management. In this report, we present an interesting case of cutaneous TB that manifested as a chronic non-healing ulcer of the foot for 15 years in a healthy middle-aged individual without any risk factors or systemic symptoms.

## Introduction

Tuberculosis (TB) is a chronic infectious disease primarily caused by the bacterium *Mycobacterium tuberculosis*. TB has been a public health concern since ancient times, and it is the leading cause of mortality due to an infectious agent. According to the World Health Organization (WHO) *Global Tuberculosis Report 2024*, the total number of TB cases in 2023 was 10.8 million, with 1.25 million reported deaths [1,2]. Although TB primarily affects the lungs, extrapulmonary TB (EPTB) infections can occur in 8.4-13.7% of cases. Cutaneous TB (CTB) is a rare form of EPTB, accounting for approximately 1-1.5% of all EPTB cases. The disease is more prevalent in TB-endemic areas and in immunocompromised individuals [1]. Moreover, it has a wide spectrum of clinical presentations and can mimic a plethora of other infectious and non-infectious dermatoses [1,3]. Cutaneous tuberculosis can spread via three main routes: exogenously through direct inoculation, endogenously from underlying infected structures, or via haematogenous dissemination. These different routes can result in a variety of clinical morphologies, ranging from a localized TB chancre to acute miliary TB presenting with multiple erythematous papules and pustules [3,4]. Other diagnostic challenges may arise due to difficulty in isolating and culturing the mycobacteria, resulting in delays in diagnosis and treatment [5]. Locally, in Oman, the TB incidence is low, with sporadic surges observed due to cross-border migration. In 2021, the TB incidence rate in Oman was 3.2 per 100,000 amongst nationals [6]. There are no documented CTB cases locally in the literature as of yet. This report highlights an interesting case of a chronic non-healing tubercular foot ulcer in a middle-aged healthy Omani male.

## Case presentation

**Figure 1:**
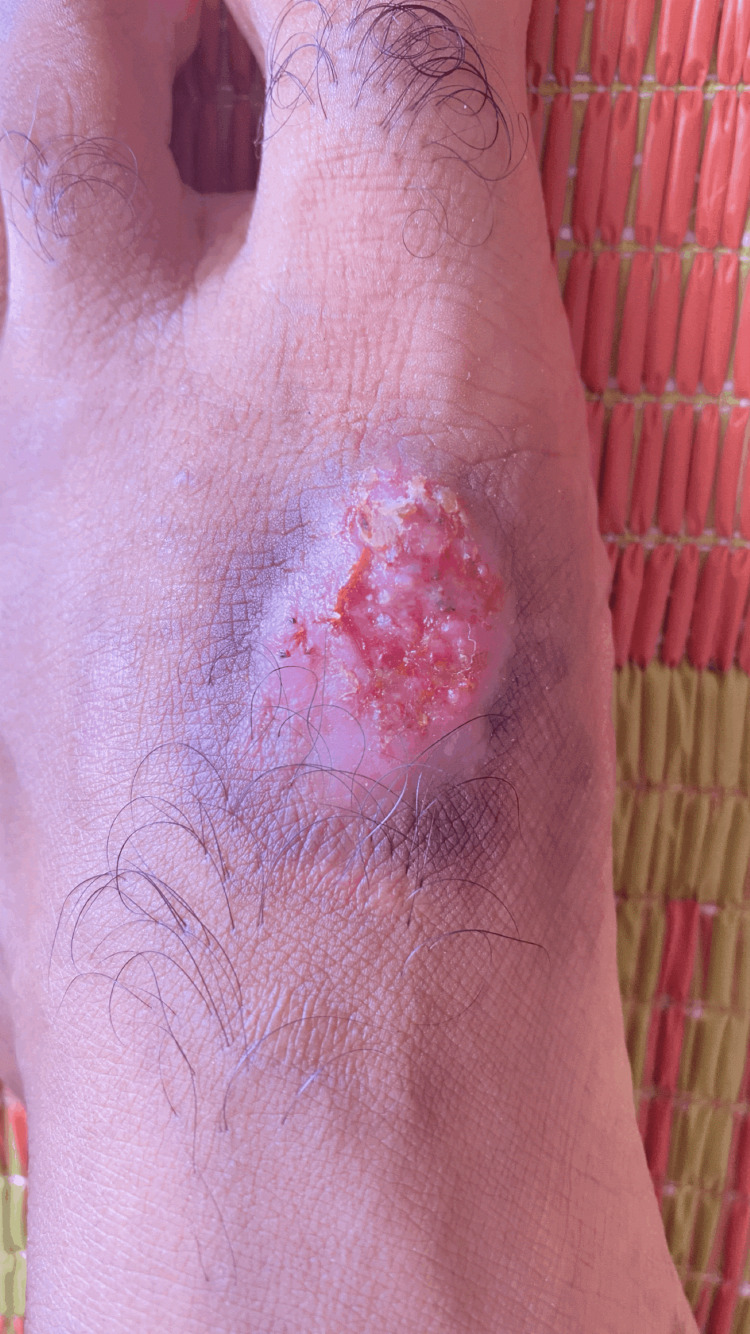
Chronic non-healing left-foot ulcer with an erythematous centre and a violaceous rim

A 36-year-old Omani male nurse presented to the dermatology clinic with a 15-year history of a non-healing left foot ulcer. His past, personal, and medical history were insignificant. The lesion started as a small erythematous papule, which later developed into a small ulcer after his colleague stepped on it while playing barefoot football. The lesion continued to increase in size and had an ulcerated, erythematous centre with a violaceous rim (Figure 1). The ulcer was itchy and easily bled on minor trauma, with exudative serous fluid occasionally. Using a topical corticosteroid would help make the lesion dry for short periods of time. At the time of presentation to our clinic. The patient was complaining of a painful oedematous erythematous ulcer over the dorsomedial aspect of the left foot. The patient was afebrile, and his vitals were within the acceptable range. Lymph nodes were impalpable. The rest of the limb examination was normal.

Review of systems was unremarkable. He denied any systemic symptoms, including fevers, night sweats, and weight loss. The patient had no other underlying medical conditions. He worked as a school nurse; the ulcer started before starting clinical rotations. He had no history of smoking, drinking alcohol, or illicit drug use, and no history of recent travel before the onset of the lesion. The patient denied any contact with animals, plants, or fresh water. His family history was insignificant for non-communicable and infectious diseases.

The lesion was initially diagnosed and treated as nummular dermatitis. Over the years, he had used multiple courses of topical and oral antibiotics, antifungals, and corticosteroids, including intralesional triamcinolone injection. Nevertheless, minimal to nil improvement was noted. The patient had few and inconsistent visits to the clinic over the years, bouncing between multiple physicians. 

Laboratory investigations yielded unremarkable results, and serologic screening for blood-borne infections was negative (Table 1).

**Table 1:** Laboratory findings

Laboratory Parameter	Result	Reference Range
Haemoglobin (g/dL)	16.9	11.5-15.9
Leukocyte Count (cells/mm3)	6200	2200-10000
Erythrocyte Sedimentation Rate (mm/hr)	5	0-15
C-Reactive Protein (mg/L)	1.7	0-10
Glucose in Serum (mmol/L)	5.09	4.1-5.9
Glycated Haemoglobin (%)	5.19	4.4-6
Estimated Glomerular Filtration Rate (ml/min/1.73m2)	>90	>90
Creatinine in Serum (umol/L)	84.14	64-104
Alanine Transaminase in Serum ([iU]/L)	31.59	0-38
Aspartate Transaminase in Serum ([iU]/L)	24.01	10-50
Hepatitis B Virus Surface Antigen	0.37	<1 (Non-Reactive)
Hepatitis B Virus Core Antibody	0.01	<1 (Non-Reactive)
Hepatitis B Virus Surface Antibody (m[iU]/ml)	>1000	—
Hepatitis C Virus Antibody	Non-Reactive	—
Human Immunodeficiency Virus 1+2 Antigen/Antibody	Non-Reactive	—
Rapid Plasma Reagin	Non-Reactive	—

**Figure 2:**
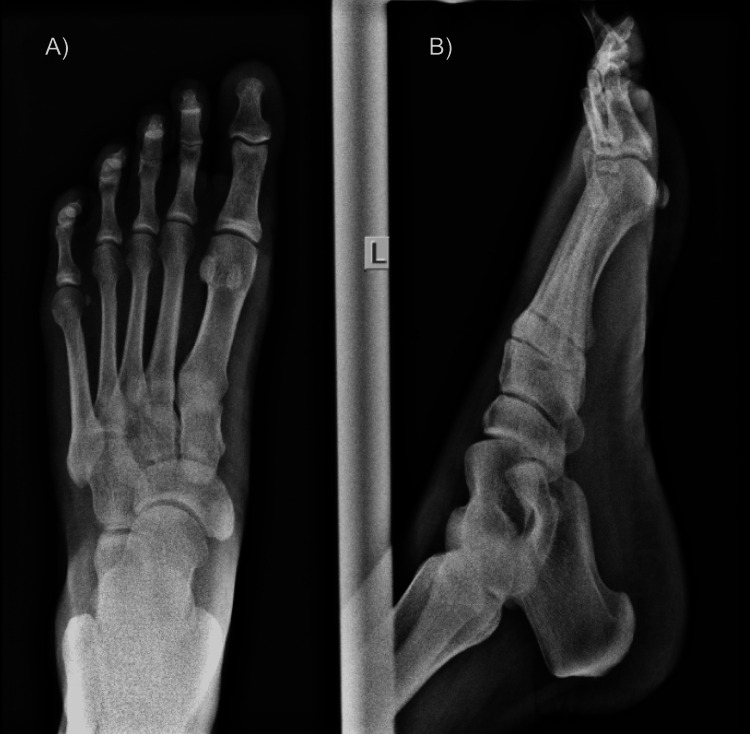
Left foot X-ray images in dorsoplanter (A) and lateral (B) views

An X-ray of the affected foot, in dorsoplanter and lateral views, was unremarkable, with no periosteal changes (Figure 2). Ultrasonography scan revealed minimal superficial subcutaneous soft tissue oedema; otherwise, no masses or abscess formation was noted.

**Figure 3:**
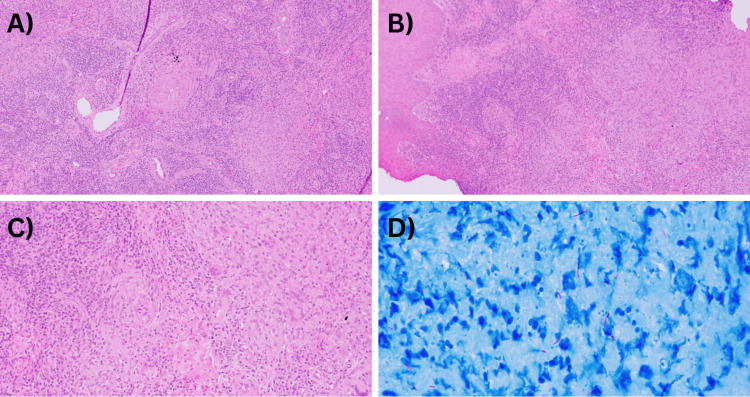
Histology findings A-C) Histopathology shows pan-dermal tuberculoid granulomas formed by epithelioid histiocytes, numerous Langerhans giant cells, multinucleated giant cells, and surrounded by numerous lymphocytes. D) Positive Ziehl-Neelsen stain

A skin biopsy was taken, and the histology findings (Figure 3) were suggestive of CTB infection. There were pan-dermal tuberculoid granulomas formed by epithelioid histiocytes, numerous Langerhans giant cells, numerous multinucleated giant cells, few plasma cells, and surrounded by numerous lymphocytes. Ziehl-Neelsen (ZN) stain revealed positive results for TB Bacilli. Also, the ZN stain from a sinus discharging pus swab tested positive for acid-fast bacilli (AFB).

**Figure 4:**
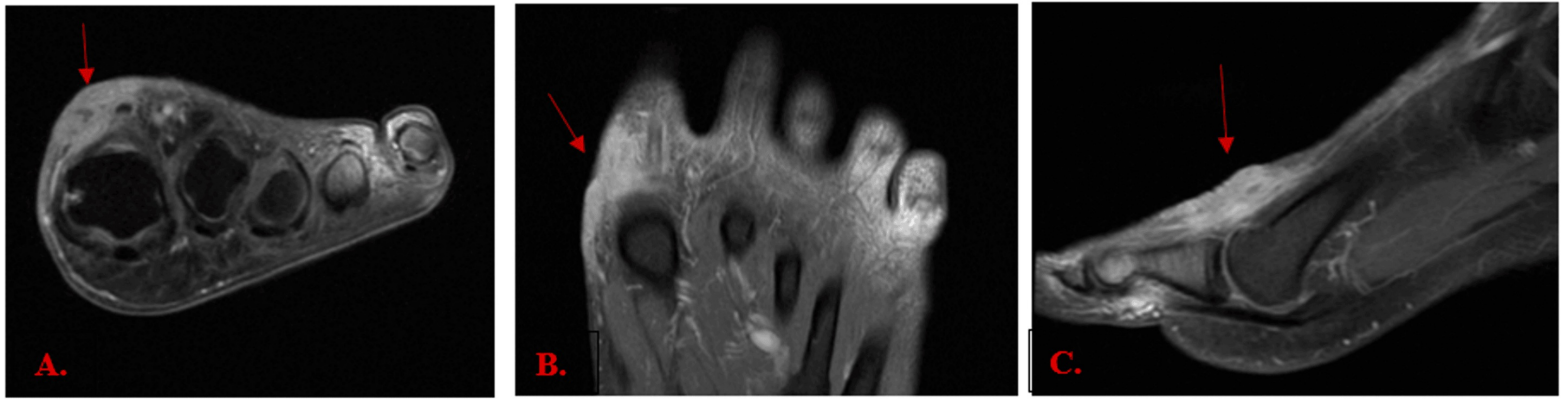
Post-contrast MRI images of the left foot Post-contrast left foot MRI images in the axial (A), coronal (B), and sagittal (C) planes showing diffuse enhancement of the dorsal skin and subcutaneous tissues (red arrows).

An MRI of the left foot was undertaken to rule out TB osteomyelitis. Post-contrast MRI images in axial, coronal, and sagittal planes showed diffuse enhancement of the dorsal skin and subcutaneous tissues, as indicated by red arrows in Figure 4, consistent with superficial inflammatory or infectious soft-tissue involvement. There was no bone marrow enhancement, and the osseous structures were preserved, thus excluding osteomyelitis.

**Figure 5:**
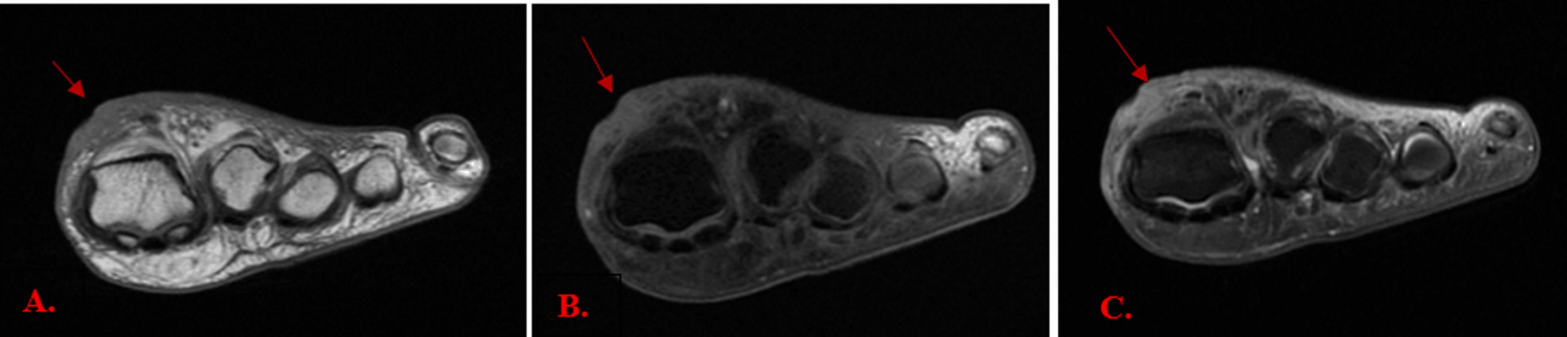
MRI scan images of the left foot in T1, T1FS and PD sequences MRI images of the left foot in the axial view in T1-weighted image (A), in T1-weighted fat-saturated (T1FS) image (B), and in proton density (PD)-weighted image (C), showing a skin ulcer at the dorsum of the great toe with surrounding reactive subcutaneous inflammation (red arrows). No evidence of osteomyelitis appreciated.

Figure 5 presents axial MRI images in T1-weighted, T1 fat-suppressed (T1FS), and proton density (PD)-weighted sequences. The scans show a skin ulcer at the dorsum of the great toe, with surrounding reactive subcutaneous inflammation, as indicated by red arrows. There was no bone marrow edema or abnormal marrow signal, and no evidence of osteomyelitis. 

**Figure 6:**
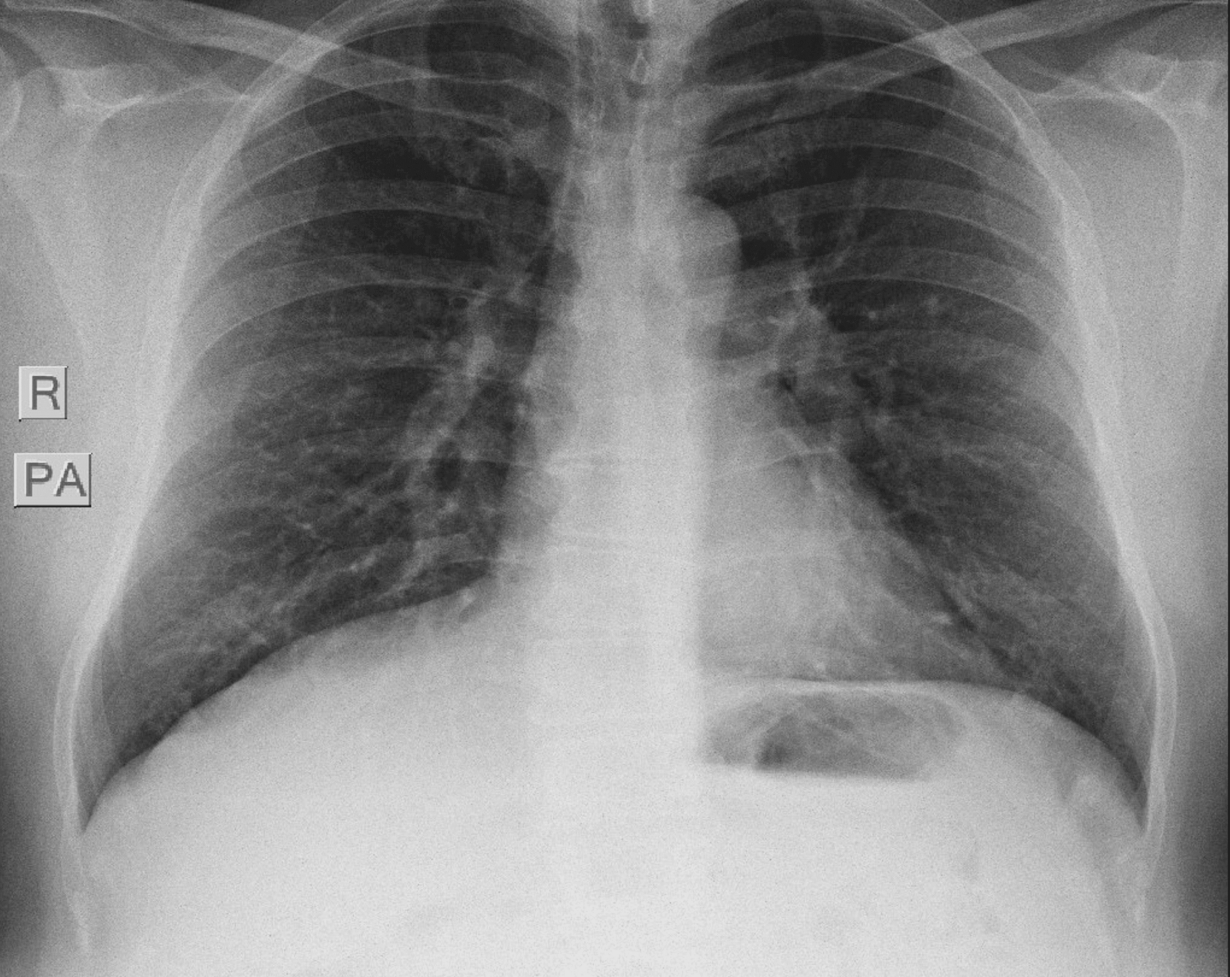
Grossly normal chest X-ray

The patient was referred to the TB clinic for further evaluation, as part of the screening process, particularly considering his occupational risk as a healthcare worker. A chest X-ray image was reported as normal (Figure 6). Sputum AFB stain, tuberculosis skin test (TST), and interferon gamma release assay (IGRA) were negative (Table 2).

**Table 2:** Tuberculosis workup IGRA: interferon gamma release assay; AFB: acid-fast bacilli

Laboratory Parameter	Result
Sputum AFB	Negative
Tuberculosis Skin Test (Mantoux test)	3 millimeters induration
IGRA	Negative

**Figure 7:**
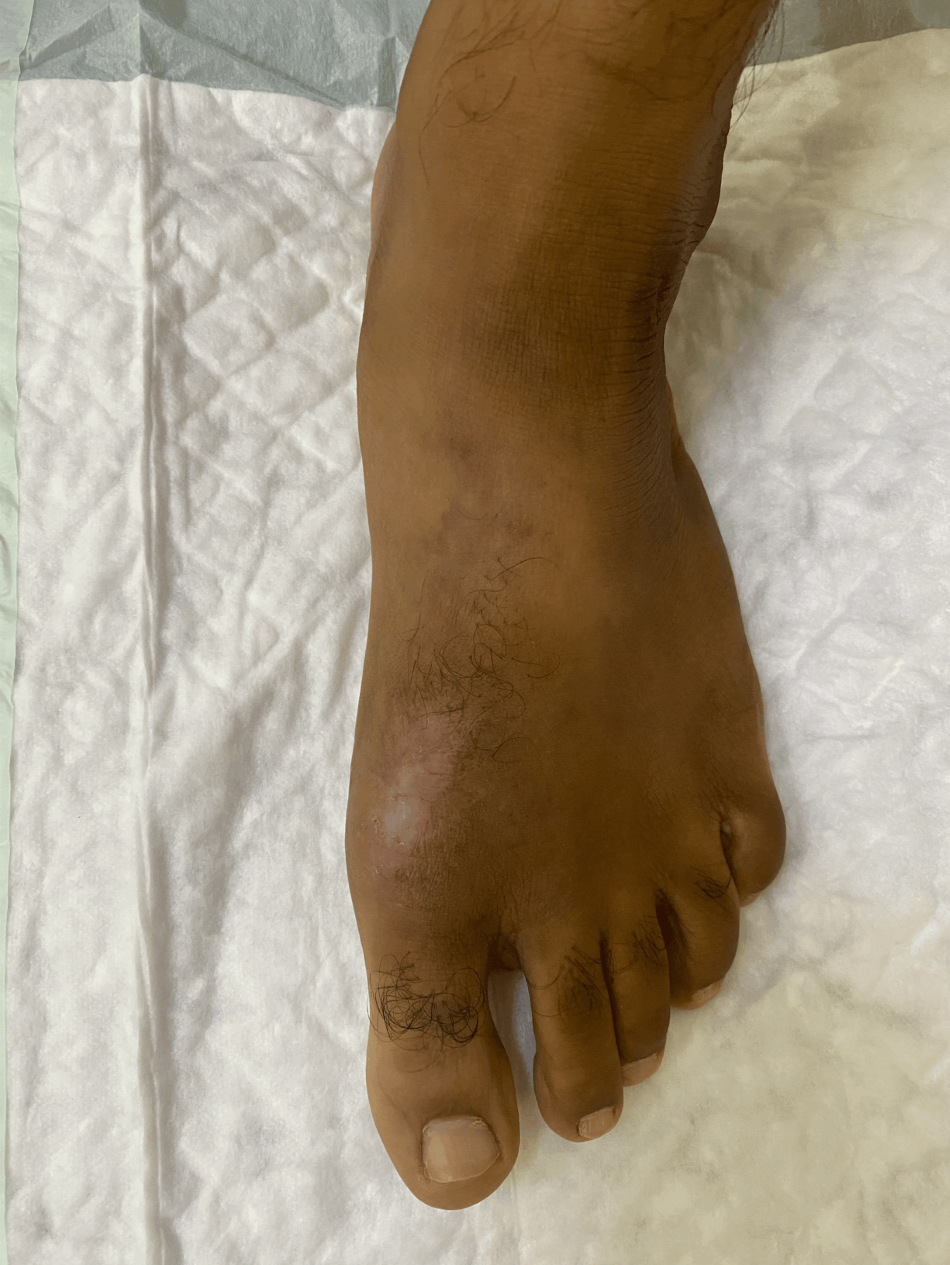
Hypopigmented atrophic scar over the dorsum of the left foot after nine months of anti-tuberculosis medications

The patient was treated with rifampicin, isoniazid, pyrazinamide, and ethambutol for two months, followed by seven months of rifampicin and isoniazid. The ulcer started to respond dramatically to the treatment, and full resolution was achieved by the end of the eighth month of treatment, leaving a hypopigmented atrophic scar (Figure 7). The patient was followed up regularly at the clinic, and there was no recurrence of the ulcer for more than one year, following completion of the anti-TB treatment.

## Discussion

Cutaneous TB is a rare entity of EPTB. The literature suggests that even in endemic areas, like India and Brazil, the prevalence does not exceed 2% of all TB cases. CTB can manifest in various forms, resulting in delays in diagnosis [3,4]. For example, lupus vulgaris presents as an erythematous-brown plaque that favours the face. Scrofuloderma is another common CTB form, which presents as a subcutaneous nodule that ulcerates and forms draining sinus tracts [3,4]. Furthermore, different forms can share overlapping features, such as ulceration. They can also morph into other CTB forms; for example, a primary TB chancre can turn into scrofuloderma or lupus vulgaris [1,3]. 

The infection can spread mainly through three routes. Exogenous spread occurs as a result of the direct introduction of the bacterium to the skin, for example, by trauma. TB chancre is transmitted via this route. Endogenous spread can occur through contiguity from a surrounding TB-infected structure, such as scrofuloderma, resulting from an underlying infection of lymph nodes or bones. Haematogenous spread is transmitted from a distant focus via the bloodstream, such as acute miliary TB [1,3,4]. Other factors, such as host immunity and exposure to TB, can influence the manifestation of CTB. Due to the complexity of CTB classification, we were unable to accurately classify the type in this case. However, it is hypothesized that the bacilli were introduced via direct inoculation due to trauma and started as a TB chancre, which eventually ulcerated and possibly turned into scrofuloderma with a draining sinus tract, an indurated plaque, and violaceous edges. 

CTB tends to disproportionately impact immunocompromised individuals, for example, Human Immunodeficiency Virus (HIV) patients. Also, lower socioeconomic status is linked to higher rates of infection, since those patients are more likely to be living in unhygienic and overcrowded conditions with poor nutritional status. Other risk factors include smoking, alcoholism, living in endemic areas, and contact with TB cases [1,3-5]

A scarce number of case reports of CTB in low-incidence TB countries can be found in the literature, and all of them are seen in individuals with a history of living in TB-endemic regions [7-9]. In our case, our patient was diagnosed with CTB despite being immunocompetent with seemingly no apparent risk factors; hence, it is crucial to consider CTB as one of the differential diagnoses of chronic non-healing ulcers in immunocompetent individuals, even in regions with low TB rates. 

A skin biopsy for histopathological examination and AFB staining is vital for the diagnosis. Tuberculoid epithelioid granulomas, caseous necrosis, and giant cells are classical histopathological findings in CTB. Along with AFB staining using special stains, such as the Ziehl-Neelsen stain [4,10], isolation of the mycobacterium in culture is the gold standard; nevertheless, this is not always feasible. Sensitivities for CTB vary significantly in reports, ranging from 0% to 62.8%. CTB can be further classified as multibacillary, which contains a high bacillary load, and paucibacillary, with a low bacillary load, the latter being more difficult to isolate and detect [1,3,10].

Moreover, TST and IGRA can aid in the evaluation of CTB, but they cannot confirm the diagnosis or distinguish active disease from latent infection or past exposure. Molecular typing, using polymerase chain reaction and nucleic acid amplification tests, is becoming increasingly popular. All suspected CTB should be screened for HIV, pulmonary TB, and any other possible EPTB manifestations. Screening examples for EPTB can include fine-needle aspiration cytology of lymph nodes and MRI of bony structures to rule out TB osteomyelitis. If all CTB workup is negative, a good response to a trial of anti-TB medication can confirm the diagnosis [1,10].

If not treated, CTB can cause disseminated TB or local tissue destruction. The first-line treatment consists of isoniazid, rifampin, ethambutol, and pyrazinamide for two months, followed by four months of isoniazid and rifampicin [5,10]. Duration can be extended up to nine to twelve months if there is extensive or deep tissue involvement [10]. In this case, the patient received a nine-month course of treatment, achieving full lesion resolution by the eighth month. Surgical intervention may be required in some cases [1]. Multidrug-resistant TB is on the rise. If the response to the treatment is inadequate, resistance testing is needed, and second-line agents should be tried [10].

Our case highlights a significant delay in diagnosing CTB, which was exceptionally lengthy in our case. In the majority of CTB case reports we found, the duration of the lesion before diagnosis did not exceed two years, even in non-endemic areas, with only one case report from Sudan having a delay of seven years [11]. Moreover, the lack of systemic symptoms and exposure to TB, normal laboratory values, and being in a non-endemic area contributed to delays and misdiagnosis with inappropriate management. This case highlights the importance of considering CTB as a differential diagnosis in atypical, non-healing ulcerative lesions in individuals with no known risk factors for CTB.

## Conclusions

Cutaneous TB is a very rare skin infection. It is notorious for its wide spectrum of manifestations and its ability to mimic a multitude of skin conditions. This report emphasizes the importance of having a high index of suspicion when encountering atypical non-healing ulcers. The rarity of CTB, living in an area with low TB incidence, and the patient’s immunocompetency should not sway the treating clinicians away from the diagnosis. CTB workup should be promptly initiated in such cases, as the disease can have a significant impact on the patient if left untreated.
